# Privacy concerns in social media UGC communities: Understanding user behavior sentiments in complex networks

**DOI:** 10.1007/s10257-023-00631-5

**Published:** 2023-03-11

**Authors:** Jose Ramon Saura, Daniel Palacios-Marqués, Domingo Ribeiro-Soriano

**Affiliations:** 1 Rey Juan Carlos University, Madrid, Spain; 2 Universitat Politècnica de València, Valencia, Spain; 3 Universitat de Valencia, Valencia, Spain

**Keywords:** Social media communities, Privacy concerns, UGC, Social network analysis, Sentiment analysis

## Abstract

In a digital ecosystem where large amounts of data related to user actions are generated every day, important concerns have emerged about the collection, management, and analysis of these data and, according, about user privacy. In recent years, users have been accustomed to organizing in and relying on digital communities to support and achieve their goals. In this context, the present study aims to identify the main privacy concerns in user communities on social media, and how these affect users’ online behavior. In order to better understand online communities in social networks, privacy concerns, and their connection to user behavior, we developed an innovative and original methodology that combines elements of machine learning as a technical contribution. First, a complex network visualization algorithm known as ForceAtlas2 was used through the open-source software Gephi to visually identify the nodes that form the main communities belonging to the sample of UGC collected from Twitter. Then, a sentiment analysis was applied with Textblob, an algorithm that works with machine learning on which experiments were developed with support vector classifier (SVC), multinomial naïve Bayes (MNB), logistic regression (LR), random forest, and classifier (RFC) under the theoretical frameworks of computer-aided text analysis (CATA) and natural language processing (NLP). As a result, a total of 11 user communities were identified: the positive protection software and cybersecurity and eCommerce, the negative privacy settings, personal information and social engineering, and the neutral privacy concerns, hacking, false information, impersonation and cookies data. The paper concludes with a discussion of the results and their relation to user behavior in digital environments and an outline valuable and practical insights into some techniques and challenges related to users’ personal data.

## Introduction

The development of the internet in the last decade has driven the adoption of digital strategies in the business ecosystem (Matt et al. 2015). In this connected ecosystem, users have been configured as key elements that increase engagement in social networks and the information management process in digital environments (Montalvo 2011). Simultaneously, users organize and participate in digital communities—i.e., virtual spaces where they interact with peers and share their common interests, tastes, opinions, and so forth (Hilvert-Bruce and Neill 2020; Martín and Fernández 2022). In this way, more and more data related users’ online behavior, organization and concerns are being generated every day (Jordan 2019; Santos et al. 2022).

In parallel, the exponential development of digital data sources has brought forward virtual crimes, digital fraud, as well as online attacks that have become a problem not only for user privacy (Krishnamurthy and Kucuk 2009). In a digital ecosystem where online communities, users, influencers, companies, and brands have to interact, there must be an appropriate definition of actions that can cause concerns about user privacy (Muniz and O’guinn, 2001; Chopra et al. 2022). Furthermore, negative experiences that users live through in digital environments can promote cybercrime actions, which may increase the number of victims of cyber-attacks (Arslan et al. 2022; Afolabi et al. 2021).

At the same time, companies use online communities to create and influence user needs so that to lead customers to purchase their products and services (Kuo and Feng 2013). As one of such strategies, leaders of digital communities, also known as micro influencers, are hired by companies to transmit a message or a commercial objective (Kucuk 2016; Yen et al. 2021). Overall, companies use online communities in digital environments and social media (Lacarcel and Huete 2023) with the following three fundamental objectives: (i) advertising objective, through which companies try to obtain economic benefits; (ii) growth objectives, through which companies try to increase the time that users spend using technology, and (iii) engagement objectives, whereby companies try to make users increasingly interact with the dynamic elements of companies in digital environments and, by doing so, influence their behavior and generate more data (Saura et al. 2021a; Chopra et al. 2022).

However, as argue by Zuboff (2015), these commercial objectives can modify user behavior and raise important privacy concerns. Accordingly, the main purpose of the present study is to identify the main privacy concerns by analyzing user communities in the social network Twitter. To date, none of the previous study has used tools that work with artificial intelligence (AI) to identify online communities and their link user privacy concerns, and therefore, contributing to increase the novelty and originality of the study (Gupta et al. 2018). Bridging this gap in the literature, and using the current knowledge about user-generated content (UGC), the present study aims to identify and understand user behavior in these digital communities based on the feelings found in each of them, as well as provide insights into user-generated behavior (UGB) (Saura et al. 2021b).

Specifically, the present study addresses the following two research questions (RQ): (RQ1) What are the main concerns about privacy in user communities on Twitter? (RQ2) How do the privacy concerns in Twitter communities affect user behavior in digital environments? In addition, this study also aims to identify perspectives to define user privacy in social network communities; to explore different types of UGC and UGB in digital communities; to create awareness regarding user privacy concerns in social media communities and to provide future guidelines to understand online user behavior social network communities.

To address the aforementioned research questions, we use an innovative and original methodology that combines the elements of AI. Concretely, following Blondel et al. (2008) and Jacomy et al. (2014), we first use the open source Gephi software and a data visualization algorithm for complex networks known as ForceAtlas2 to visually identify the nodes that form the main communities belonging to the analyzed sample. Second, based on the database of tweets divided into communities, a sentiment analysis is run with Textblob (Khan et al. 2021), an algorithm that works with machine learning where experiments are developed with support vector classifier (SVC), multinomial naïve Bayes (MNB), logistic regression (LR), and random forest classifier (RFC) (Hiremath and Patil 2020). The approaches used in this study fit the theoretical framework of computer-aided text analysis (CATA) (Short et al. 2010) and natural language processing (NLP) (Hirschberg and Manning 2015).

## Literature review

Sharma et al. (2020) proposed the idea that users’ demographic and psychographic variables can influence their engagement in digital communities. Furthermore, Ortiz (2019) argued that user use online communities to obtain information related to their interests, segment the content they enjoy in digital environments, as well as pursue professional activities (Sahoo and Gupta 2020). At the same time, in an ecosystem where more and more data are generated online, the feeling of belonging to an online community and the motivations why users become part of such communities are directly linked to users’ personality and behavior (Liu et al. 2016).

In this respect, Bouguessa and Romdhane (2015) demonstrated that, though being part of an online community, users feel authorized to share their ideas. Similarly, online communities can either accept or reject user ideas and behaviors, which leads to the establishment (or lack therefore) of certain digital activities, behaviors or ideologies (Williams and Cothrel 2000). Furthermore, in their daily lives, users can be affected by actions linked to malicious activities such as cyberbullying or cyberattacks (Carter 2013). However, belonging to a digital community that supports the same interests will allow a user to obtain information related to his/her interests and assumer digital roles that would help his/her personal causes and goals (Almomani et al. 2022).

Several previous studies linked online communities to privacy and the collaborative economy, since online communities have become platforms that can exchange products and services among equals and bring together a multitude of users (Kordzadeh et al. 2016). Furthermore, privacy has also been linked to trust in the production of digital actions and the relationships between users in online communities and their behaviors (Fogel and Nehmad 2009).

In this context, grounded theory must be highlighted. This theory advocates that researcher systematically select qualitative content to build hypotheses or theories through data collection and has also been used to structure research studies related to privacy (Roseman and Smith 2001). Also, appraisal theory is used as a theory that argues that elicited emotions as evaluations of mass events can cause specific reactions in people. Therefore, our appraisal of a situation causes an emotional event that responds to that appraisal. In this way, these types of events are found in digital communities and linked to the emotions of users according to the topics that are analyzed (Youn 2005; Deuker 2012).

In this way, digital communities can be a valid data source for the development of this type of study. As argued by Kitsios et al. (2022), online communities can be the beginning of companies’ data mining analysis to analyze and collect user behavior and pursue what is referred to as online behavior modification (Di Caprio et al. 2022). As noted by Zuboff et al. (2019), using the analysis and influence of online communities, companies seek to create economic stimuli to increase the profitability of their advertising and try to collectively and massively modify user behavior (Saura et al. 2022a).

The insights that companies extract using AI-based strategies and algorithms to collect and analyze data can be very profitable. These actions can cause addiction among consumers (Ribeiro-Navarrete et al. 2021; Saura 2021), thus promoting the creation of more data that improve both the algorithms working with machine learning and their results over time, as well as encourage the creation of increasingly solid digital communities (Franco and Esteves 2020).

In addition, the use of big data in the analysis of online communities can encourage companies to develop strategies that are unethical with respect to user privacy (Muhammad et al. 2018). Similarly, users’ lack of knowledge about the power of AI, as well as uncertainty as to whether their data are collected individually or collectively, can raise important concerns among users about treatment, collection, and development of predictions related to their online behaviors (Kafeza et al. 2019).

In this connection, Kim (2016) highlighted user privacy concerns in relation to their location. In fact, user geographic information can be an extremely rich source of information for companies when it comes to improving their products and conducting market research on the international level (González-Padilla et al. 2022). In this context, in the present study, we identify and extract knowledge using the technologies presented above so that users, scientists, companies, and practitioners can better understand the importance of ethical design of data collection and analysis, as well as strategies based on active listening to online communities.

## Methodology

### Data sampling

The data extraction and analysis process has followed the procedure previously proposed by Hiremath and Patil (2020). Specifically, we connected to the public Twitter API to download pieces of UGC in the form of tweets with the hashtag #Privacy #PrivacyConcerns. The queries containing the indicated hashtags were made from May 5 to May 15, 2022. These dates were used because there was no international event linked to user privacy or similar issues that could have changed the results (Kim et al. 2013).

The terms used to collect the data from Twitter matched our research questions and objectives (Saura et al. 2022b). For the development of the data collection, a non-probability sampling frame was followed (Schillewaert et al. 1998). As indicated in Saura et al. (2022c), “non-probability sampling is an approach to sample elaboration, which is also known as simple judgment” (p. 3). In this study, we followed the criteria formulated by Lehdonvirta et al. (2021) and Saura et al. (2022c) to develop the sampling process in which we considered non-probabilistic variables. Lehdonvirta et al. (2021) indicated that non-probability sampling is commonly used in studies that work with UGC from social networks.

The data collection process was developed with Mac version of Python 3.7.0. Once the data were collected, the tweets were filtered. First, images and videos were excluded, since this study focused on NPL, thus not covering visual or interactive elements of the database. This is a common process in studies that analyze Twitter data (Rafail 2018). In addition, links and URLs were removed from the database in order to clean the content. Additional filtering processes were applied using Python and Pandas libraries. Only those tweets that belonged to UGC online communities on Twitter were analyzed, taking into account the ties and connections existing in the users and the content generated around the community.

Similarly, repeated tweets were eliminated, as were also emojis to enhance the quality of the data. In this way, from the database of 75,307 tweets, after the filtering process and identification of communities, a total UGC sample of 49,701 tweets was obtained.

### Social media network analysis

To apply data visualization algorithms and online communities, we used the algorithm developed by Blondel et al. (2008). This algorithm makes it possible to identify communities in complex networks through the modularity report (MR) indicator. This technique uses a resolution enhancer to improve visualization of the algorithm based on Lambiotte et al. (2014). This type of algorithm used to visually represent online communities was previously used by Jacomy et al. (2014).

**Fig. 1:**
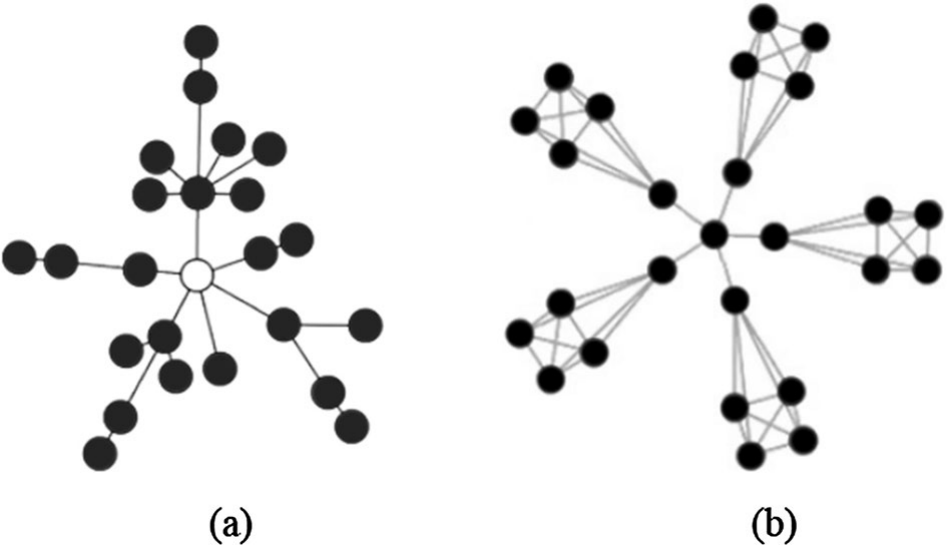
Node connections among Twitter UGC communities (*Source*: Adapted from Saura et al. (2019))

In the present study, the main objective of the identification of online communities was to understand the role played by privacy concerns of users and their online behavior. In this way, our sample was structured into nodes related to each other. Nodes are neurons that represent links between different users and which, when analyzed together, form communities on Twitter (Tidke et al. 2020) (see Fig. 1a, b). Figure 1a shows the global community of users linked by the nodes according to their connections. Figure 1b shows these in an enlarged way, thus identifying sub-communities that make up the global community and that determine topics of conversations, interests, and comments.

The link between these nodes and the relevance and weight they have over the remaining nodes is what determines their relationships in terms of relevance in the total database (Java et al. 2007). Accordingly, the nodes indicate the communities and the themes that compose them. UGC communities in social media can be visually represented both by the number of users who make up a node and the number of topics that make up the set of nodes in an online community (Barbosa et al. 2022; Guerola-Navarro and Stratu-Strelet 2022).

As concerns the resolution of the algorithm and its application, it tries to identify the sub-communities based on the weight of nodes linked to the main community, which, in the present study, is that of online privacy concerns. According to Ausserhofer and Maireder (2013), the higher the visualization and its resolution when the algorithm is applied, the fewer but more specific communities are obtained (Gu et al. 2022). Conversely, the lower the resolution, the more communities, as well as the more users and their links are identified by the algorithm (Chawra and Gupta 2022).

As demonstrated by Fogel and Nehmad (2009), networks of nodes can be visually understood as systematically organized communities around the union of nodes. The organizational utility of this type of system is that it analyzes the links between each node and their distance in terms of weight (Trott 2022). One node can be identified in relation to several descriptors, which makes it possible to demonstrate the themes around which the communities are grouped, as well as their classification. The richer in terms of data a community is, the more connections are there between its nodes (Mishra et al. 2011; Lam and Chow 2022).

### Sentiment analysis

As noted by Tokarchuk et al. (2022), there are different approaches to divide a database into different sentiments. In the present study, we used the theoretical framework of NLP and CATA, which is a sentiment analysis of text (i.e., excluding visual and multimedia elements in a network). Following Hiremath and Patil (2020) and Saura et al. (2022b), sentiment analysis was performed using the Textblob algorithm (Rasmusen et al. 2022; John and Sam 2022). This algorithm, developed in Python with NLTK and patterns, has been widely used in the literature.

However, a limitation of sentiment analysis is that it fails to identify connotations or sarcasm, as well as irony (Hussein 2018). The Textblob algorithm was trained, and these issues were taken into account in order to proceed with an efficient and accurate development of the results. In order to develop the algorithm with high precision, the tweets were rated based on their polarity and subjectivity. Polarity was rated from − 1 to 1 and subjectivity from 0 to 1.0 (Hiremath and Patil 2020; Saura et al. 2022b).

The algorithm was trained a total of 935 times using tweets that had been manually classified (Neethu and Rajasree 2013). That is, manually classified tweets were used as inputs. Upon learning, the algorithm classified the remaining tweets into three groups depending on the sentiment expressed in them (positive, negative, and neutral). As argued by Taboada (2016), feelings can be extended to other emotions or moods. However, following Hiremath and Patil (2020), in the present study, we decided to focus on the analysis of only three aforementioned feelings. Similarly, aiming to increase precision of our sentiment analysis development process, five cross-validation experiments were performed (Saura et al. 2022b; Mohammed et al. 2022).

Specifically, precision, recall, f-1 score, and support were used as measurement factors and variables. Sentiment analysis results were obtained in terms of macro average and weighted average. The models used as experiments in the 5 cross validation experiments were as follows: support vector classifier (Sarkar et al. 2019), multinomial naïve Bayes (Griol et al. 2017), logistic regression (Sarkar et al. 2019), and random forest classifier (Vijayarani and Janani 2016).

## Results

### Social media network analysis

Using the approach proposed by Blondel et al. (2008) to the sample, we identified user communities with different resolutions. The objective was to configure the largest number of relevant user communities found in the sample in relation to privacy and user behavior. In order to obtain the best results, four different tests were performed applying the algorithm in different resolutions. The results were interpreted based on the number of communities, modularity report, modularity resolution, maximum modularity class, and minimum modularity class (Bouarara 2021).

The modularity report indicates the relevance and weight of the identified community: the higher the modularity, the higher the relevance of a community in the database. Modularity resolution indicates the resolution of the algorithm used to visualize the communities. Maximum and minimum modularity class indicates the maximum and minimum relevance value identified in the total of the identified communities (Blondel et al. 2008). The results of five tests that were performed are shown in Table 1.

**Table 1:** Modularity report communities’ results by test (*Source*: The authors)

Test	Number of communities	Modularity	Maximum modularity class	Minimum modularity class	Resolution used	Modularity resolution
1	1082	0.006	110	0	0.001	− 0.009
2	731	0.009	126	0	0.01	− 0.013
3	201	0.092	135	0	0.1	− 0.29
4	73	0.227	140	0	0	0.136
5	25	0.281	144	0	1	0.187

**Fig. 2:**
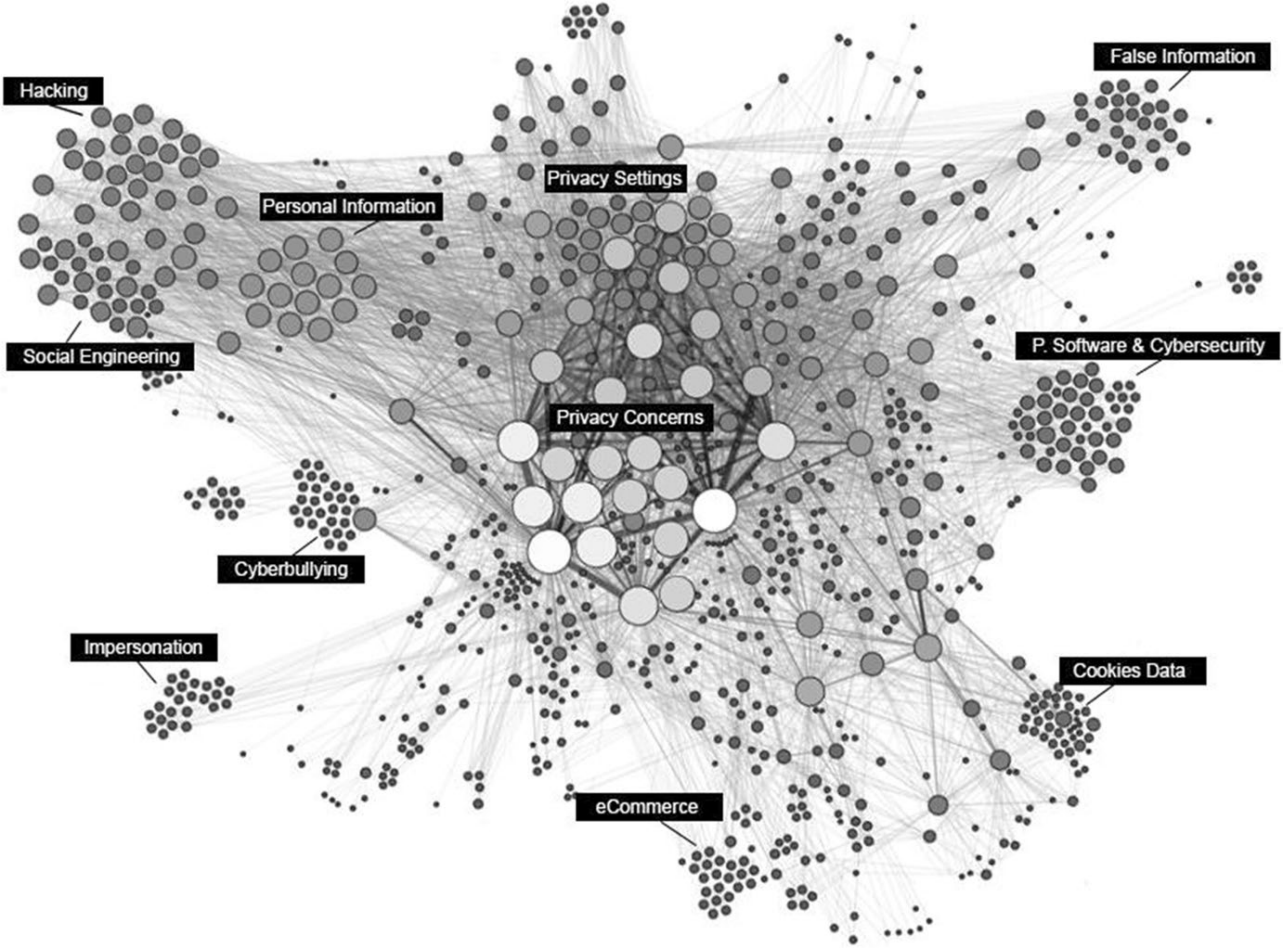
Communities related to privacy identified in Twitter UGC (*Source*: The authors)

Aiming to improve the visualization of the neural network and the identified communities, several filters were implemented so as to enlarge the resolution of the identified communities using Gephi 0.9.7. Following Jacomy et al. (2014), in order to modify the variable modularity report and the algorithm in charge of visualizing the data, we used Force Atlas and Force Atlas2. These filters can be applied with Gephi software. The following data visualization filters were applied: threads number (1), tolerance (1.0), approximation (0.1), scaling (0.2), gravity (1.0), prevent overlap (True) and edge weight influence (4.0). The size of the resolution in relation to the visualization of each node was 10 points. The number of nodes analyzed were 9860 and 141,551 edges. Dynamic graph and multi graph attributes were not applied. The type of graph was undirected using auto-scale. The edges merge strategy was sum. The total time consumed to apply Force Atlas and Force Atlas2 algorithm before they were stopped were 3 min. The nodes were then grouped into communities in relation to their weight in the identified community and were displayed in different colors. In terms of weight in the MR, the communities were organized as follows: privacy concerns (MR = 140), privacy settings (MR = 136), personal information (MR = 116), hacking (MR = 113), social engineering (MR = 110), protection software (MR = 107), false information (MR = 101), cyber-bulling (MR = 97), impersonation (MR = 96), cookies data (MR = 96), and eCommerce (MR = 94). In is important to notice that the Fig. 2 has been graphically adapted to show the identified communities accordantly. In Fig. 2, the identified communities are shown. The size of each community is represented by the number of nodes that compose it. The distance between each community as well as the distance to the center of the neural network, determine the relationship and the proximity of the topic to privacy concerns.

### Sentiment analysis

Next, we performed sentiment analysis. The accuracy variable was identified as the metric that verifies the quality of the sentiment analysis process (Sharma et al. 2021). In the present study, the accuracy variable was used to measure the effectiveness of algorithms that work with machine learning. The five technologies used to validate the analysis are presented in Table 2 with Sl. No. from 0 to 4. The table shows the number of the experiments, the name of the model used, the fold_idx value that represents the order of the text and, finally, the accuracy obtained in Textblob (Alowibdi et al. 2021).

**Table 2:** Accuracy results of sentiment analysis according to the experiments

Sl. No	Model name	Fold_idx	Accuracy–Textblob
0	Random forest classifier	0	0.780872
1	Random forest classifier	**1**	0.773359
2	Random forest classifier	2	**0.790031**
3	Random forest classifier	3	0.781021
4	Random forest classifier	4	0.783631
5	Linear SVC	0	0.878403
6	Linear SVC	1	0.868901
7	Linear SVC	2	**0.880057**
8	Linear SVC	3	0.859021
9	Linear SVC	4	0,805,321
10	Multinomial naïve Bayes	0	0.800511
11	Multinomial naïve Bayes	1	0.805850
12	Multinomial naïve Bayes	2	0.810825
13	Multinomial naïve Bayes	**3**	**0.800979**
14	Multinomial naïve Bayes	4	0.800712
15	Logistic regression	0	0.876001
16	Logistic regression	1	0.879129
17	Logistic regression	2	0.874239
18	Logistic regression	**3**	**0.869138**
19	Logistic regression	4	0.863008

Bold values indicate highest scores obtained for accuracy in each experiment

In line with Hiremath and Patil (2020) and Saura et al. (2022b), the highest accuracy value obtained was in relation to linear SVC Sl. No.7 (0.880057). For RFC, the results were Sl. No.2 (0.790031). As concerns LG, the precision of 0.800979 was obtained in Experiment 3. Finally, with regard to multinomial naïve Bayes, the highest result was Sl. No. 4, with the accuracy value of 0.800979.

To summarize and facilitate the understanding of the results of the sentiment analysis, Table 4 presents the names of the models used to develop the sentiment analysis with Textblob and the highest scores obtained for accuracy in each case. As can be seen in Table 3, the accuracy of LinearSVC was 0.880057 the highest and was the used for the development of the study.

**Table 3:** Summarized brief scores of Textblob analysis

Sl. No	Model name	Scores of Textblob analysis
1	Linear SVC	0.880057
2	Logistic regression	0.869138
3	Multinominal NB	0.800979
4	Random forest classifier	0.790031

Furthermore, it is a standard procedure in research carried out with NLP and CATA to represent the variables of recall, f1-score, and support (along with accuracy). In the present study, the sentiment analysis algorithm was configured around three sentiments (positive, negative and neutral). The indicated variables were therefore linked to each of these feelings (Chen et al. 2022). As argued by Lease (2011), the accuracy variable refers to the quality of the machine-learning algorithm, while the recall variable measures the amount of machine learning (such as technology) used to support the development of the model applied to the database of tweets.

Furthermore, the f1-score variable is a metric that combines both the accuracy and recall variables in a single analytical indicator (Heikal and Eldawlatly 2020; Singh and Sachan 2021). Therefore, using f1-score makes it possible to compare different behaviors of the algorithm in relation to the accuracy of the model used. Similarly, the support variable determines how much machine learning is used by the model to make the predictions with which the accuracy is computed. Table 4 presents these variables, as well as the weighted average measures associated with relativity in terms of weight.

**Table 4:** Sentiment analysis classification report using Textblob

Sl. No	Parameters	Vader			
		Precision	Recall	f1-score	Support
1	Negative	0.87	0.86	0.86	20.399
2	Positive	0.88	0.84	0.87	22.510
3	Neutral	0.83	0.79	0.76	20.529
4	Accuracy	–	–	0.83	43.161
5	Macro avg	0.86	0.85	0.82	43.161
6	Weighted avg	0.83	0.83	0.82	43.161

In order to be able to divide the identified communities into sentiments, all tweets in the database linked to each of the identified communities were selected and divided into different databases. In this way, once the tweets of the communities in different databases were extracted, the sentiment analysis was applied to the set of tweets contained in these databases. Table 5 lists the identified communities, their descriptions, and the associated sentiments.

**Table 5:** Communities identified, description, sentiment and precision, MR and its weight (*Source*: The authors)

No	Community name	Description	Sentiment/precision	Modularity report/weight
1	Privacy concerns	General issues related to privacy concerns on digital environments	Neutral (0.83)	140 (1)
2	Privacy settings	Main initiatives to improve the settings of digital devices in relation to the increase in security and data breaches	Negative (0.87)	136 (2)
3	Personal information	Comments and experiences linked to access to user personal information through social networks; negative evaluation of metadata, access and transfer of user data	Negative (0.87)	116 (3)
4	Hacking	Comments on ethical hacking or criminal hacking viewed upon as technical knowledge, rather than as criminal actions in digital environments	Neutral (0.83)	113 (4)
5	Social engineering	A multitude of negative experiences in relation to cases where social engineering led to extortion, identity theft, or unauthorized sale of user data	Negative (0.87)	110 (5)
6	Protection software and cyber security	Possibilities for improving security and the risks of using technological devices in social networks; a multitude of protection and antivirus software alternatives, as well as commercial messages from companies where they sell this software	Positive (0.88)	107 (6)
7	False information	Information and debates related to fake news and leaking of false information on social networks; a discussion of the great technological advances that companies have made to detect this type of information and block it in real time	Neutral (0.83)	101 (7)
8	Cyberbullying	Recent experiences and case studies linked to cyberbullying actions; ideas on how to counteract these events in the context where the development of technology is exponential, and the institutions are no longer capable of adapting their protocols at the same speed	Negative (0.87)	97 (8)
9	Impersonation	Discussion on the impossibility of identifying real people behind social network profiles; problems caused by phishing actions; proposals of new technologies to verify profiles on social networks and demonstrate that there are people behind their configuration and published content	Neutral (0.83)	96 (9)
10	Cookies data	Information and discussion regarding how the content on social networks is linked to cookies collected by technological devices and specifically browsers	Neutral (0.83)	96 (10)
11	eCommerce	The strategies developed by hackers to illicitly obtain user information in online purchases; positive experiences of recovering stolen money or data; a discussion of the growing increase in security by companies, such as the implementation of secure access protocols	Positive (0.88)	94 (11)

## Discussion

The results of using our innovative methodology helped us identify a total of 11 user communities where users discussed and commented various privacy concerns. As argued by Jozani et al. (2020), privacy concerns have become one of the main topics in current research on digital ecosystems. Indeed, digital communities and social networks are a great source of information for identifying problems and understanding the behavior of online users (Ribeiro-Navarrete et al. 2021).

The positive community that we identified was entitled protection software and cyber security (MR de 107(6)); in this community, the main focus was on the issues related to the improvement of security initiatives in digital inmates. In addition, technological devices and social networks were discussed as the main channels that make users install (and developers create) protection and security software that allows blocking of illegal or illicit actions. Users modify their online behavior based on the perceived security of the devices they use. In this respect, we agree with Baldassarre et al. (2019) who argued that the development of protection and security software should be increased in the future to increase user protection.

Furthermore, in the second positive community, eCommerce (modularity report by 94(11)), we identified insights related to the strategies developed by hackers to illegally obtain credit card information databases or perform other illegal actions. However, in this community, users shared positive experiences of how they managed to recover their money owing to the actions of banks and financial companies. As noted by Mohammed and Tejay (2017), e-commerce platforms have considerably increased security of their payment platforms and gateways by adding security protocols and channels such as HTTPs. This allows users to confidently navigate in digital environments and feel safe (Harridge‐March, 2006).

The first among the negative communities that we identified was privacy settings (modularity report of 136(2)). In this community, users discussed the main initiatives to improve settings and configurations of digital devices. According to Ketelaar and Van Balen (2018), digital devices are the main access channels to obtain personal data from users who use those devices on a daily basis. However, when establishing connection to public internet networks, privacy settings of these devices can adversely affect users. According to Saura, privacy on these devices should be established by default (Saura et al. 2021c). This concept would allow users not to adapt their online behavior depending on the security of their devices. This would ensure privacy by default once the product is purchased for the first time.

Another negative community in our results was personal information (modular report of 116(3)), which contained a multitude of comments and experiences linked to events when personal information was obtained from users in a lawful manner. Indeed, as indicated by Park (2013), some users can unknowingly share their personal information that can later be used by cyber criminals for unlawful purposes. In this respect, we agree with Lacárcel (2022) who argued that educational programs should be developed to train users on how to properly manage their personal data.

The third negative community identified in our results was social engineering (modularity of 110(5)). In this digital community, users discussed the main techniques that cyber criminals develop to make users give up their personal data, images, financial or health information that may then be illegally sold to interested third parties. As indicated by Saura et al. (2021b), these types of actions are common in social networks where users publish content without being aware of the value of their data and personal information that can be collected from this type of content. Finally, the last negative community was cyberbullying (modularity of 97(8)) where users shared recent experiences and case studies linked to cyberbullying actions, as well as discussed ideas on how to counteract these events.

Furthermore, among five neutral communities identified in the present study, privacy concerns (modularity of 140 (1)) dealt with general issues related to privacy concerns in the use of both social networks and digital platforms. Both positive and negative experiences were discussed and shared. In general, users share current and future concerns linked to the massive use of digital applications. However, as argued by Pal et al. (2021), the society connected through IoT devices can cause privacy concerns, as users might not be aware of the management of their data and the insecurity of certain actions they perform on a daily basis. Accordingly, their behaviors must be adapted to the type of social network or platform they use.

The second neutral community was false information (modularity of 101(7)). In this community, false content known as fake news was mainly commented and shared. In this connection, Zhang and Ghorbani (2020) showed that fake news increased in the COVID-19 pandemic (Rodríguez-Priego and Porcu 2022), as well as during other international events that evoked global interest in access to information. One of the main purposes of publishing false information is to increase visits to the published content so that companies can get higher returns from their digital advertising (Yin and Lin 2022). Blom and Hansen (2015) linked these actions to the concept of easy click, or clickbait, which can be monetized and thus bring financial benefit to companies.

The third neutral community was impersonation (modularity of 96(9)). In this community, users discussed the problem of identifying real people from their social network profiles. As indicated by Gordon et al. (2007), identity theft remains to be a big problem in social networks, as there are duplicate and false profiles. Accordingly, a reasonable idea, proposed by Brandtzaeg et al. (2016), is that profiles on social networks should be publicly verified so that users can be sure that there is a real person behind the content disseminated in a social network.

The fourth neutral community was cookie data (modularity of 96(10)) where users and discussed how the adoption of browsers and technological programs on computers allows the collection of more and more cookies linked to their personal data. According to Stoycheff (2022), this can also be one of the ways for the generation of personalized and intelligent content that makes users worry about their personal information and digital behavior. This type of personalized actions, according to Arora and Bawa (2022), can make users feel that their data are used fraudulently. However, users increasingly understand this type of practice and are aware of such actions when they interact with this technology. Finally, the fifth neutral community was Hacking (modularity of 113(4)). In this digital community, users commented on ethical hacking or criminal hacking viewed upon as technical knowledge, rather than as criminal actions in digital environments.

### Theoretical implications

The results of the present study can be used by other researchers to explore new challenges and opportunities in the industry of privacy in digital environments and user behavior. Specifically, in future studies, the identified communities can be used as variables and/or constructs of empirical and quantitative models that could be empirically tested. In addition, the communities identified through the algorithm of complex social networks ForceAtlas2 allow other researchers to replicate these techniques in future studies.

Finally, the sentiments associated with the identified communities identified and the weight of each community can help other researchers to set more specific research objectives to address opportunities and issues related to privacy in a more specific and detailed way.

### Practical implications

As concerns practical implications, this study provides valuable and practical insights into some techniques and challenges in digital environments related to users’ personal data. Similarly, by understanding the communities involved, general users and practitioners, as well as digital agencies and public institutions linked to digital business models, can better understand how users share their opinions on the Internet and organize themselves specifically in Twitter.

Accordingly, our results can help both companies to develop their strategies and individuals to form and develop safer browsing habits. Likewise, the results of this study can be used to improve the security and communication protocols of public institutions that are dedicated to the education of healthy navigation habits. Institutions such as the United Nations or the European Commission in Europe can use the results identified to increase investment in programs to educate on security on social network.

## Conclusions

In the present study, we used an innovative methodology that works with machine learning to extract insights from digital communities on Twitter related to privacy and online user behavior. Based on our results, a total of 11 user communities were identified: two positive (protection software and cyber security and eCommerce), four negative (privacy settings, personal information, social engineering, and cyberbullying), and five neutral (privacy concerns, hacking, false information, impersonation, and cookies data).

As concerns our research questions, in relation to RQ1 (*What are the main privacy concerns in the user communities on Twitter?*), we identified 11 such communities and established which sentiments (positive, negative, or neutral) they are associated with. Furthermore, as concerns RQ2 (*How do the privacy concerns of Twitter community users affect their behavior in digital environments*?), we discussed the communities identified in relation to the behavior of users on social networks and platforms.

Of note, however, users can adapt their behavior in digital environments according to their perceptions of privacy and trust in each digital ecosystem. At the same time, some users lack skills for the appropriate management of their personal data, which can lead to security breaches. In addition, users should be aware of the impact of their actions in digital environments and social networks, as well as the information contained in the publications and content they generate in digital ecosystems.

In order to protect themselves from possible hacking actions, users should understand how social engineering strategies work and acquire knowledge. Similarly, acquisition and adoption of new protection software that increases the security of user data must be installed on user devices. Furthermore, proper identification of false information and awareness of impersonation of certain profiles on social networks can help users avoid cases of cyber bullying. Similarly, the correct use of cookies and the collection and analysis of data can increase the protection and security of electronic commerce platforms in the medium term. In summary, a proper understanding of the communities’ topics identified in the present study will allow users to feel safer in online communities and avoid typical risks.

### Limitations and future research

Limitation of the present study are related to the number of tweets in our sample, as well as to the application and filtering according to the resolution used in the neural network identification algorithm used in Gephi. In the future, in order to increase precision of the results, the same research design could be applied to a larger sample and improved algorithms based on machine-learning technology.

## Data Availability

The datasets generated and analysed during the current study are not publicly available due the fact that they constitute an excerpt of research in progress but are available from the corresponding author on reasonable request.
